# Effects of dietary glycerol, vitamin C and niacinamide supplementation on liver of growing-finishing pigs

**DOI:** 10.3389/fvets.2025.1620128

**Published:** 2025-06-18

**Authors:** Wenchen Sun, Linglan Deng, Wanjie Zou, Panting Wei, Shaobin Hao, Huadong Wu, Wei Lu, Yuyong He

**Affiliations:** ^1^Jiangxi Province Key Laboratory of Animal Nutrition and Feed, Engineering Research Center of Feed Development, Jiangxi Agricultural University, Nanchang, China; ^2^College of Animal Science and Technology, Jiangxi Agricultural University, Nanchang, China

**Keywords:** liver, microbiota, RNA sequence, histomorphology, glycerol, vitamins, pig

## Abstract

The influence of supplementing glycerol, vitamin C and niacinamide on the liver of growing-finishing pigs has not yet been examined. This study investigated the effect of 10% glycerol, 0.06% vitamin C and 0.05% niacinamide supplementation at single or combination on liver of growing-finishing pigs. Compared with pigs supplemented with 0% glycerol, 0% vitamin C and 0% niacinamide, pigs supplemented only with 10% glycerol had higher (*p* < 0.05) TNF-*α* concentration, partially hepatic steatosis, higher (*p* < 0.05) relative abundances of *Escherichia_shigella*, *Prevotellaceae_UCG_003*, *Lachnospiraceae_XPB1014_group*, *Coprococcus*, *Lactococcus* and *Megamonas*, lower (*p* < 0.05) solute carrier family 7 member 11 (*SLC7A11*) expression in liver tissue. However, pigs offered the diet with a mixture of 0.06% vitamin C and 0.05% niacinamide had higher (*p* < 0.05) relative abundance of *Faecalibaculum* and expression of *SLC7A11*, lower (*p* < 0.05) relative abundances of *Staphylococcus* and *Clostridium_sensu_stricto_1* in liver tissue. Supplementation of 10% glycerol, 0.06% vitamin C and 0.05% niacinamide simultaneously to pigs increased (*p* < 0.05) the ferrous ion level, the relative abundances of *Escherichia_Shigella*, *Lactococcus* and *Desulfobacterota*, the expressions of gene Cryptochrome-1(*CRY1*) and *SLC7A11*, but decreased (*p* < 0.05) the expressions of gene C-reactive protein (*CRP*) and galactokinase 1 (*GALK1*) in liver tissue. Supplementation with 0.06% vitamin C and 0.05% niacinamide can alleviate the damage in liver of pigs fed a diet containing 10% glycerol.

## 1 Introduction

The liver is a very important organ in the body of animals, because it has vital functions in metabolism, secretion, detoxification and immunity (1–3). Failure of hepatic cells to functions is often associated with the inappropriate intake of ingredients in the diet by the development of steatosis, inflammation, fibrosis, or poisoning in liver tissue, which are harmful to the health and performance of animals (4–8).

Glycerol addition can enhance food palatability (9, 10) as well as provide energy (11). It is reported that the metabolism of glycerol largely occurs in liver and it can induce anatomical, physiological, and biochemical changes in the liver when glycerol is included in the food (12, 13). Currently glycerol is one of the most promising ingredients in formulating the diet of animals, owing to it can be used as an alternative energy source to corn in the diet (14), and also some studies reported that inclusion of glycerol to the diet can improve animal performance, meat quality and gut health by sparing the catabolism of dietary amino acids (15–17). However, information about the effect of long-term glycerol intake on the liver of animals is scarce.

Niacinamide and vitamin C are often added to the diet of animals. Niacinamide serves as the precursor of coenzyme I [nicotinamide adenine dinucleotide (NAD)+/NADH] and coenzyme II (NADP+/NADPH) with the participation in glycolysis, tricarboxylic acid cycle, fatty acid synthesis and oxidation, cholesterol synthesis, and gluconeogenesis in redox reaction (18, 19). It also plays a crucial role in lipid metabolism, adipocyte differentiation, post-transcriptional modification, and inflammation inhibition in non-redox reactions (18, 20, 21). Dietary administration with niacinamide from 20 to 640 mg/kg dry matter (DM) decreased the contents of glucose and triglyceride, as well as fat accumulation in liver (1). However, administering niacinamide at a dose of 500 mg/kg body weight (BW) to healthy animals induced hepatotoxicity (22). Vitamin C is a strong antioxidant and it can prevent lipid peroxidation, necrosis, inflammation and fibrosis of liver by scavenging free radicals (23, 24). In addition, Vitamin C can improve meat color by inhibiting phospholipid oxidation and reactive oxygen formation, and increase marbling score and meat quality by enhancing adipocyte differentiation (25, 26). It is reported that addition of vitamin C at a dose of 10 g/steer * d to Angus-cross steers receiving the high S diet significantly increased marbling score and backfat thickness when compared to steers without vitamin C supplementation (27). Vitamin C also has regulatory effect on proinflammatory cytokine secretion in lipopolysaccharide (LPS)-challenged blood mononuclear cells of healthy human (28). Vitamin C administration displayed some protective effects on LPS-induced liver injury with a level at 200 mg/kg BW (29) and reversed hepatotoxicity at 5 mg/mL when combined with niacinamide (30).

Long-term consumption of high level of energy-generating ingredients such as fat, sugar and glycerol has the risks to deteriorate liver functions (31). Niacinamide and vitamin C are the promising candidates in ameliorating high energy consumption-induced liver diseases. Our previous study indicated that addition of 10% glycerol together with 0.06% vitamin C and 0.05% niacinamide significantly increased the muscle redness of *longissimus dorsi* of finishing pigs crossbred by Duroc, Large White and Landrace (DLL). However, the potential effects of dietary supplementation with 10% glycerol, 0.06% vitamin C and 0.05% niacinamide on the liver and pancreas of DLL pigs are still unclear. In this study, conventional and omics approaches were performed to explore the effects of dietary supplementation with 10% glycerol, 0.06% vitamin C and 0.05% niacinamide on histomorphology, microbiome and trancriptome of liver, and the findings of this study will provide a novel insight into the healthy production of pigs fed glycerol-supplemented diets.

## 2 Materials and methods

### 2.1 Feeding experiment

A total of 84 weaned piglets (20.35 ± 2.14 kg, Duroc × Large White × Landrace) were allotted to groups A (0% glycerol+0% vitamin C + 0% niacinamide), B (10% glycerol), C (0.06% vitamin C + 0.05% niacinamide), and D (10% glycerol+0.06% vitamin C + 0.05% niacinamide) at random, each group had three pens and each pen had 7 piglets. All piglets were given access to feed and water ad libitum during the 103-day feeding experiment, and the composition and nutrient level of diet are listed in Table 1 (15). All experimental procedures were conducted in accordance with the Guidelines in the Care and Use of Animals and were approved by the Ethics Committee of Jiangxi Agricultural University (JXAULL-202215).

**Table 1:** Diet composition and nutrient level^*^ (%, on dry matter basis).

Items	Group A		Group B		Group C		Group D	
	10–60 kg	60–120 kg	10–60 kg	60–120 kg	10–60 kg	60–120 kg	10–60 kg	60–120 kg
Ingredients								
Corn	59.00	64.00	48.00	52.00	58.89	63.89	47.89	51.89
Wheat bran	16.00	16.00	15.00	15.00	16.00	16.00	15.00	15.00
Soybean meal	14.00	10.00	15.00	11.00	14.00	10.00	15.00	11.00
Fishmeal	2.00	0.00	2.00	0.00	2.00	0.00	2.00	0.00
Rapeseed meal	5.00	6.00	6.00	8.00	5.00	6.00	6.00	8.00
4% Premix	4.00	4.00	4.00	4.00	4.00	4.00	4.00	4.00
Glycerol	0.00	0.00	10.00	10.00	0.00	0.00	10.00	10.00
Vitamin C	0.00	0.00	0.00	0.00	0.06	0.06	0.06	0.06
Niacinamide	0.00	0.00	0.00	0.00	0.05	0.05	0.05	0.05
Total	100.00	100.00	100.00	100.00	100.00	100.00	100.00	100.00
Nutrient levels								
Metabolizable energy (MJ/kg)	12.15	12.24	12.26	12.31	12.15	12.24	12.26	12.31
Crude protein	17.30	15.10	17.34	15.18	17.22	15.05	17.36	15.21
Crude fiber	3.84	3.68	3.69	3.78	3.74	3.71	3.47	3.63
Ether extract	3.81	3.68	3.43	3.50	3.94	3.88	3.57	3.65
Calcium	0.79	0.65	0.82	0.70	0.73	0.69	0.78	0.63
Total phosphorus	0.67	0.54	0.63	0.55	0.69	0.59	0.66	0.60
Lysine	1.09	0.84	1.09	0.86	1.09	0.84	1.09	0.86
Methionine + Cystine	0.57	0.44	0.56	0.43	0.57	0.44	0.56	0.43

^*^Levels of metabolizable energy, lysine, methionine and cystine are calculated values. Levels of crude protein, crude fiber, ether extract, calcium and total phosphorus are measured values.

### 2.2 Sampling of liver tissue

A total of 3 pigs were randomly selected from each pen (1 pig/pen) for each group, slaughtered after 12 h of fasting via bleeding with electrical stimulus, then, livers were taken out. After weighing, samples were taken from the left external lobe of liver. The average bodyweight of pigs slaughtered in each group was 106.17 ± 8.39 kg (group A), 106.00 ± 1.41 kg (group B), 102.33 ± 10.21 kg (group C), and 109.17 ± 7.53 kg (group D), respectively.

### 2.3 Fat measurement of liver tissues

Samples of liver were freeze-dried overnight at −50°C, then, ground into powder with sieve (100 μm). The crude fat content of samples was determined using Soxhlet extraction.

### 2.4 Histomorphological observation

Liver samples were fixed firstly in 4% paraformaldehyde overnight at 4°C, dehydrated and embedded with paraffin, then sliced into sections with a thickness of 4 mm. Sections were stained with hematoxylin and eosin and finally observed by light microscopy (NIKON ECLIPSE E100, Japan).

### 2.5 Oil-red O staining

Frozen samples were embedded in optimal cutting temperature compound (Sakura Finetek Japan Co., Ltd., Tokyo), sliced into section with a thickness of 10 μm using a cryostat microtome (HM525 NX U, Thermo Scientific, United States), and stained with oil red O. Images were obtained by a NanoZoomer S360 Digital Slide Scanner C13220-01 (HAMAMATSU PHOTONICS, Hamamatsu city, Japan), the image processing software Image J FIJI (open source, NIH, Bethesda, MD, United States) was used to measure the area of lipid droplets. The IntDen of the lipid droplets regions in sections were calculated by using Image J Fiji. The relative area of lipid droplets = the average IntDen of each treatment group/the average IntDen of the control group.

### 2.6 Measurement of iron ions and inflammatory markers in liver tissues

The levels of ferric ion and ferrous iron in the liver samples were determined with a ferrozine-based colorimetric assay (32). Concentrations of interleukin 10 (IL-10), tumor necrosis factor-*α* (TNF-α) and C-reactive protein (CRP) in the liver samples were determined using ELISA kits (Shanghai Enzyme-linked Biotechnology Co. Ltd., Shanghai, China) following the manufacturer’s protocols.

### 2.7 16S rDNA sequencing

Bacterial genomic DNA was extracted from liver samples using the CTAB (cetyl trimethyl ammonium bromide) method (33). The extracted DNA was quantified by a NanoDrop 1,000 spectrophotometer (Thermo Fisher Scientific, Pittsburgh, PA, United States) and stored at −20°C for use. The primers 341F (5’-CCTAYGGGRBGCASCAG-3′) and 806R (5’-GGACTACNNGGGTATCTAAT-3′) were used to amplify the qualified DNA of samples by targeting the V3-V4 regions. PCR amplifications were carried out in 30 μL reactions containing 15 μL of Phusion High-Fidelity PCR Master Mix, 0.2 μM of forward primer, 0.2 μM of reverse primer and 10 ng of template DNA with the following conditions: initial denaturation at 98°C for 1 min, followed by 30 cycles of denaturation at 98°C for 10 s, annealing at 50°C for 50 s, elongation at 72°C for 30 s, and finally, 72°C for 5 min (34).

The polymerase chain reaction (PCR) products were purified using magnetic beads. After purification, the PCR products were quantified with Qubit 3.0 Fluorometer (Invitrogen, United States) to generate amplicon libraries. The libraries were pooled in equimolar amounts and paired-end sequenced (PE250) at Novogene Co., Ltd. (Beijing, China) on an Illumina NovaSeq 6,000 platform (Illumina, San Diego, CA, United States). Bioinformatics analysis was conducted according to a previously published method (34). Raw data of 16S rDNA sequencing have been submitted to the database of Sequence Read Archive of the National Center for Biotechnology Information with the BioProject ID PRJNA1164983.

### 2.8 RNA sequencing and data analysis

Trizol kit (Invitrogen, Carlsbad, United States) was used to extract the total RNA of liver samples according to the manufacturer’s instructions. The integrity of isolated RNA was assayed using the RNA Nano 6,000 Assay Kit on the 2,100 Bioanalyzer (Agilent Technologies, Palo Alto, United States). RNA-Seq libraries were constructed using NEBNext® Ultra™ RNA Library Prep Kit for Illumina® (NEB, United States) according to the manufacturer’s instructions. After quality inspection through touch q-PCR system CFX96 (BIO-RAD, CA, United States), the prepared RNA-Seq libraries were sequenced on NovaSeq 6,000 platform (Illumina, San Diego, CA, United States) by Novogene Co., Ltd. (Beijing, China). The raw reads were filtered and trimmed using fastp (version 0.19.7). The obtained clean reads were aligned to reference genome using HISAT2. Fragments Per Kilobase of transcript per Million mapped fragments (FPKM) with Cufflinks were used to calculate the expression levels of each gene, and the read count of each gene were generated by HTSeq. DESeq 2 was used to identify the differentially expressed genes (DEGs) with *p*-value < 0.05 and |log2 Fold change (logFC)| > 1. KEGG pathway enrichment analysis was carried out to explore the functions of DEGs. Raw data of RNA sequencing have been submitted to the database of Sequence Read Archive of the National Center for Biotechnology Information with the BioProject ID PRJNA1055880.

### 2.9 Reverse transcription-quantitative polymerase chain reaction

Total RNA of liver samples was isolated using TransZol Up Plus RNA Kit (Transgen, China) and evaluated for the concentration and quality of RNA using NanoDropND1000 Spectrophotometer (Thermo Fisher Scientific, Madison, WI). The qualified RNA samples were reverse transcribed with TransScript Uni All-in-One First-Strand cDNA Synthesis SuperMix for qPCR (TransGen Biotech, Beijing, China). PerfectStart Green qPCR SuperMix (TransGen Biotech, Beijing, China) was used for qPCR using an ABI QuantStudio 5 systems (Thermo Fisher Scientific, United States). The primers information for qRT-PCR is listed in Table 2 and glyceraldehyde 3-phosphate dehydrogenase (GAPDH) served as the reference gene. The 2-ΔΔCt data method was used for analysis.

**Table 2:** Sequences of primers for qPCR.

Gene	Forward primer (5’to3’)	Reverse primer (5’to3’)
*GAPDH*	TGGAAAGGCCATCACCATCT	ATGGTCGTGAAGACACCAGT
	GACATCAAGAAGGTGGTGAAGCA	GTCGTACCAGGAAATGAGCTTGA
*SLC7A11*	AAAACCATCCCCCTTGCGAT	AGACAGCAAACACACCACCA
*CRY1*	AATACGGAGTCCCCTCACTGG	GGCTAGCAAGCAGGGAATTTG
*CRP*	CACTGTCTATGCTGGTGGGA	GGATGTGAGAGCCTGTGTTCA
*GALK1*	TGAGCTGTCCTGAGCTGGAT	CCAAAGCCACCACCTGTCAT

## 3 Results

### 3.1 Change of liver index, iron ions and inflammatory markers

Data in Table 3 indicated that pigs from group C and D had the highest and the lowest liver index respectively, but no significant difference was found in liver index when compared one group to the other groups (*p* > 0.05). In addition, glycerol, the mixture of vitamin C and niacinamide or interaction between glycerol and mixture of vitamin C and niacinamide did not impose significant effect on liver index of pigs from each group (*p* > 0.05).

**Table 3:** Changes of liver index, iron ion and inflammatory markers when supplemented different levels of glycerol, vitamin C and niacinamide to pig.

Items	Group A	Group B	Group C	Group D	SEM	*p*-value		
						Glycerol	Vitamin C + Niacinamide	Glycerol× (Vitamin C + Niacinamide)
Liver index (g/kg BW)	15.71	15.37	16.99	14.73	0.61	0.813	0.346	0.480
Fat (%)	17.21	18.85	17.74	18.05	0.43	0.858	0.289	0.473
Iron ion (μg/g)	50.42^ab^	42.18^b^	48.57^ab^	63.86^a^	3.12	0.478	0.070	0.038
Fe^2+^ (μg/g)	12.33^b^	11.82^b^	14.86^ab^	19.39^a^	1.09	0.201	0.008	0.119
Fe^3+^ (μg/g)	48.09	30.36	33.71	44.47	2.87	0.411	0.794	0.138
IL-10 (pg/g)	147.10	139.14	151.48	158.27	3.72	0.937	0.125	0.327
TNF-α (pg/g)	210.28^b^	239.56^a^	212.98^b^	222.78^ab^	3.76	0.005	0.271	0.133
CRP (mg/g)	31.68	34.59	35.33	26.86	1.58	0.358	0.494	0.081

Group A: 0% glycerol + 0% vitamin C + 0% niacinamide; Group B: 10% glycerol + 0% vitamin C + 0% niacinamide; Group C: 0% glycerol + 0.06% vitamin C + 0.05% niacinamide; Group D: 10% glycerol + 0.06% vitamin C + 0.05% niacinamide. Means within a row followed by different lowercase letters differ significantly (*p* < 0.05). *p*-value means the effect of glycerol, (Vitamin C + Niacinamide) or the interaction of glycerol and (Vitamin C + Niacinamide) on the liver index within the same row.

Pigs from group B had a lower level of total iron ion in liver than pigs from groups A, C and D (*p* < 0.05), respectively. Pigs from group D had higher Fe^2+^ level in liver than pigs from groups A and B (*p* < 0.05), respectively. The TNF-*α* levels of liver of pigs from groups B and D were higher than that of pigs from groups A and C (*p* < 0.05), respectively.

### 3.2 Histomorphological alterations in liver

**Figure 1:**
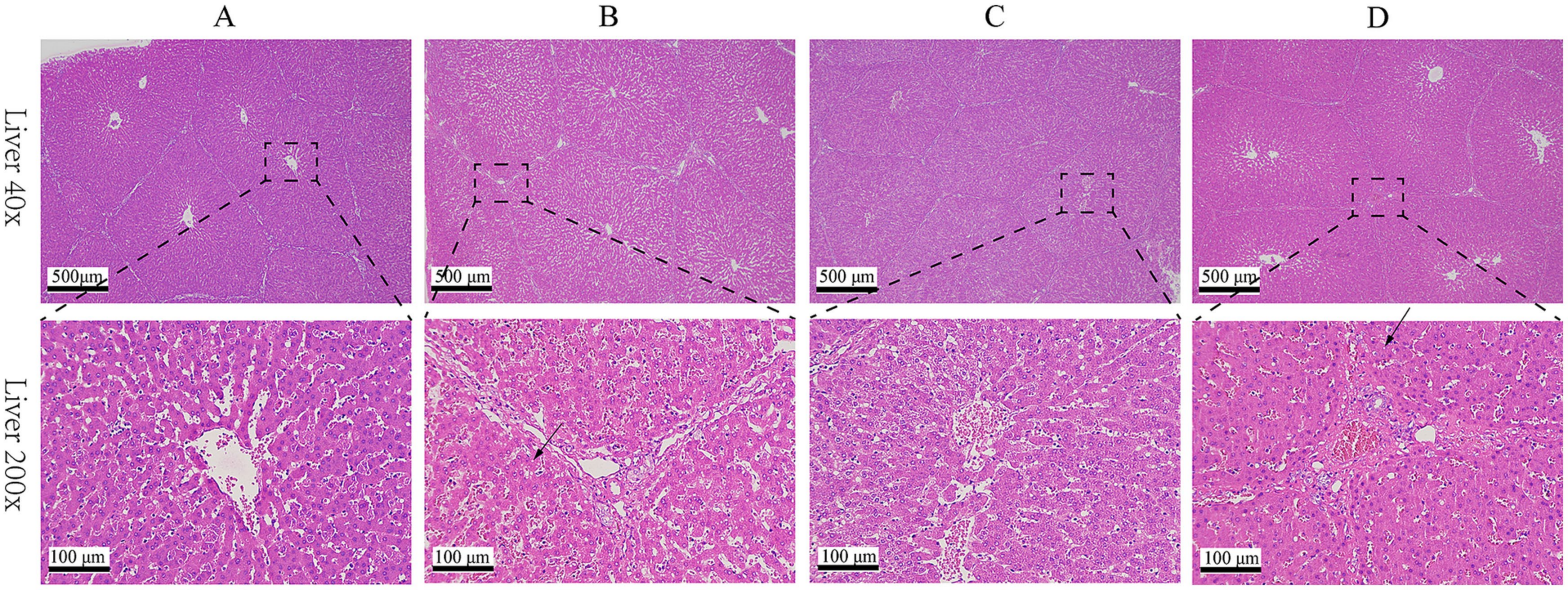
HE staining of liver tissue. **(A)** 0% glycerol + 0% vitamin C + 0% niacinamide. **(B)** 10% glycerol. **(C)** 0.06% vitamin C + 0.05% niacinamide. **(D)** 10% glycerol + 0.06% vitamin C + 0.05% niacinamide.

**Figure 2:**
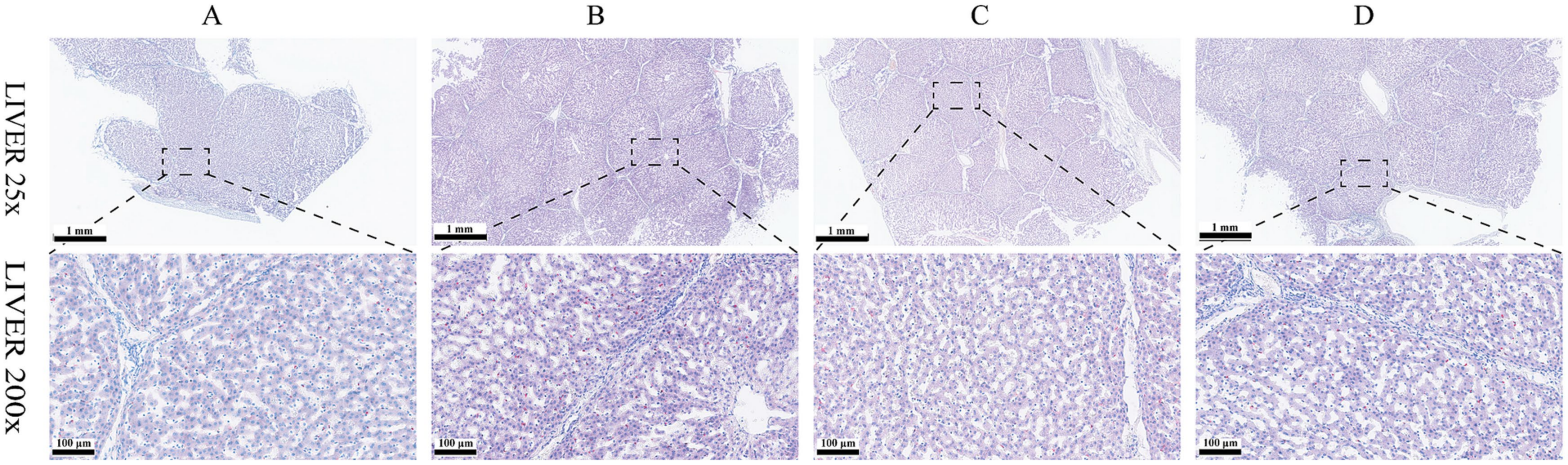
Oil-red O staining of liver tissue. **(A)** 0% glycerol + 0% vitamin C + 0% niacinamide. **(B)** 10% glycerol. **(C)** 0.06% vitamin C + 0.05% niacinamide. **(D)** 10% glycerol + 0.06% vitamin C + 0.05% niacinamide.

**Figure 3:**
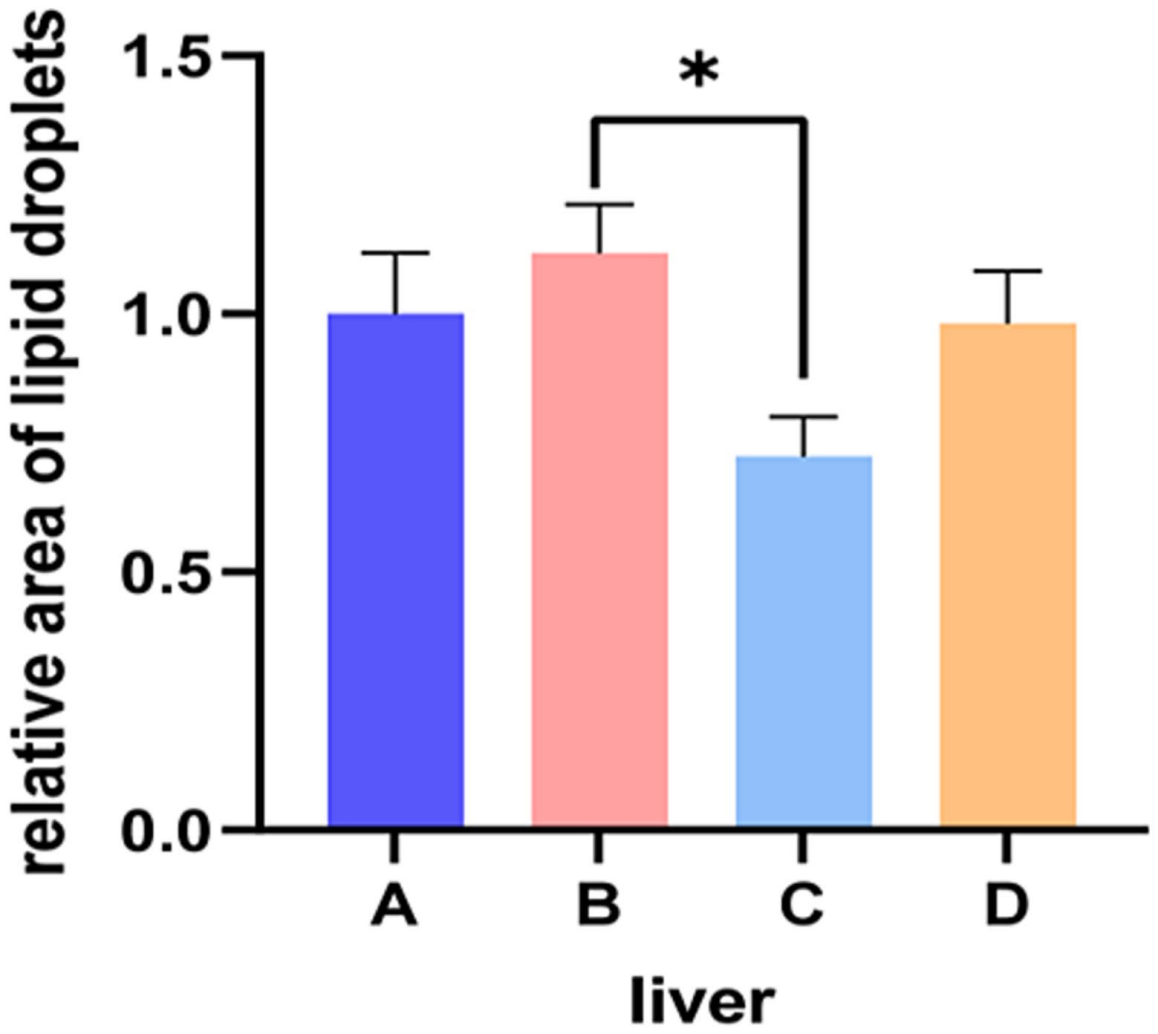
Relative area of lipid droplets in liver tissue. A, 0% glycerol + 0% vitamin C + 0% niacinamide. B, 10% glycerol. C, 0.06% vitamin C + 0.05% niacinamide. D, 10% glycerol + 0.06% vitamin C + 0.05% niacinamide. *p = 0.022.

Results of HE staining showed that the structures of liver lobules of pigs from different treatment groups were clear and intact (Figure 1). Pigs from group B presented partially hepatic steatosis (black arrow) along with small vacuoles in the cytoplasm. Pigs from group D also developed a small amount of hepatic steatosis (black arrow), but the degree of steatosis in liver of pigs from group D was less than that of pigs from group B. No obvious changes were observed in liver histomorphology of pigs between groups A and C. Addition of glycerol or/and the mixture of vitamin C and niacinamide had different effects on the accumulation of lipid droplets in hepatocytes (Figure 2), and the data of oil-red staining in Figure 3 revealed that the significant difference in relative area of lipid droplets was only found in the livers of pigs between group B and group C (*p* < 0.05).

### 3.3 Diversity and composition of bacteria in the liver of pigs

**Figure 4:**
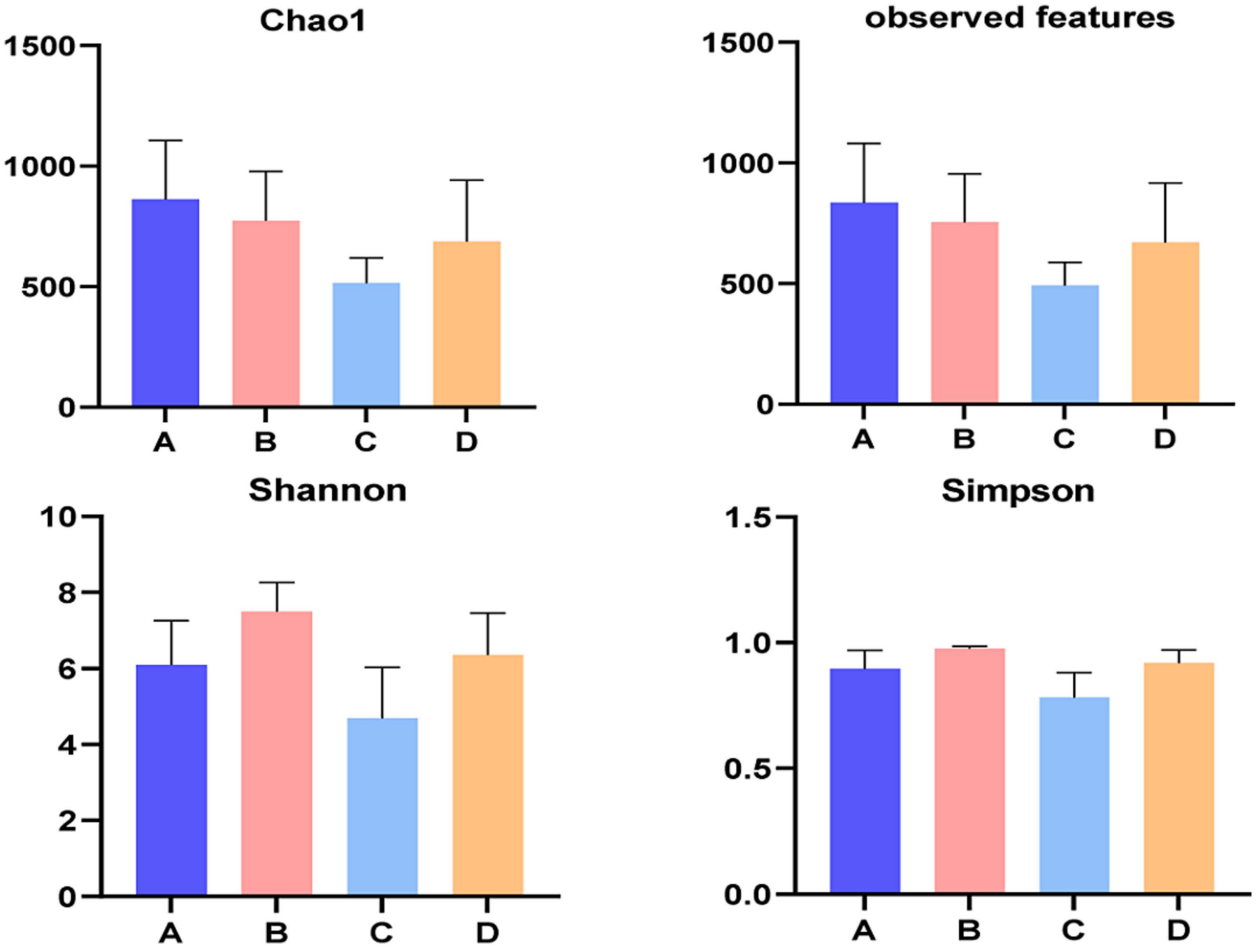
Alpha diversity of liver bacteria. A, 0% glycerol + 0% vitamin C + 0% niacinamide. B, 10% glycerol. C, 0.06% vitamin C + 0.05% niacinamide. D, 10% glycerol + 0.06% vitamin C + 0.05% niacinamide.

**Figure 5:**
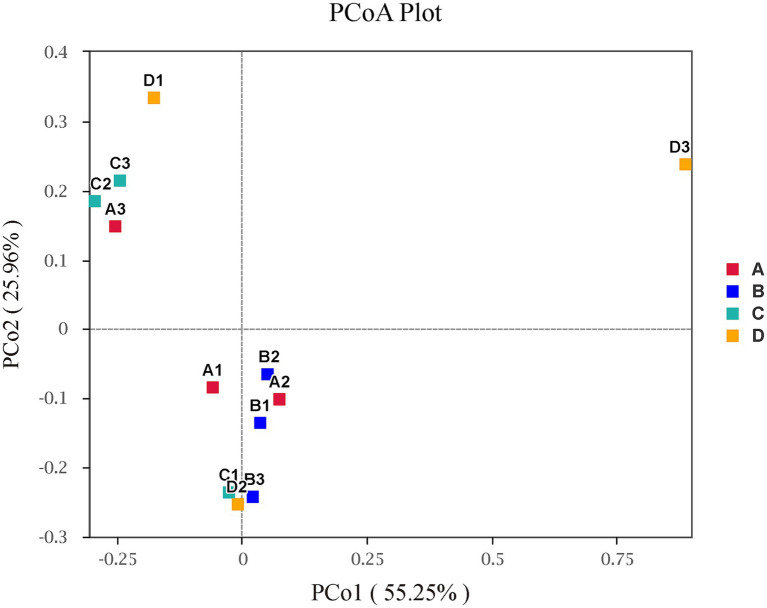
Principal coordinate analysis (PCoA) on beta-diversity with weighted-unifrac. Plots of the principal coordinate analysis (PCoA) of bacteria in liver. Samples in the same group are represented by the same color and shape. The x-axis and y-axis represent the first and second primary coordinates, respectively. The percentage in round brackets of the axis represents the proportion of the sample distance matrix that the corresponding axis can interpret. The distance between sample points indicates the similarity of bacterial communities in the samples, and the closer the sample points are to each other, the more similar the two samples are. A, 0% glycerol + 0% vitamin C + 0% niacinamide. B, 10% glycerol. C, 0.06% vitamin C + 0.05% niacinamide. D, 10% glycerol + 0.06% vitamin C + 0.05% niacinamide.

Figure 4 illustrated the alpha diversity indexes, and there were no significant differences among four groups in alpha diversity indexes of liver bacterial community of pigs (*p* > 0.05), but Chao 1 index numerically decreased (*p* > 0.05) when supplementing 0.06% vitamin C-0.05% niacinamide mixture or 0.06% vitamin C-0.05% niacinamide-10% glycerol mixture to growing-finishing pigs. The beta diversity analysis was performed to investigate the structural variation of bacterial communities. The PCoA results in Figure 5 indicated that there were no obvious intragroup aggregation except for samples in group B, and the separation between groups was not statistically significant. The PCo1 and PCo2 explained 55.25 and 25.96% of the differential contribution rate, respectively.

**Figure 6:**
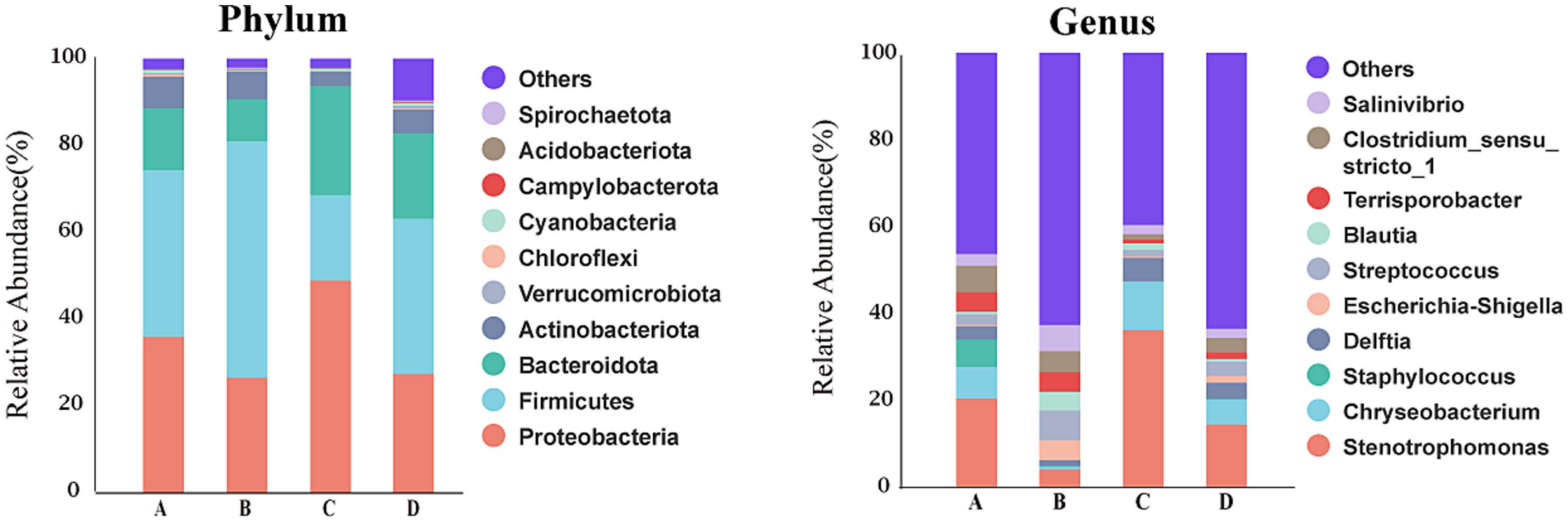
Bacterial composition of liver at different taxa levels. A, 0% glycerol + 0% vitamin C + 0% niacinamide. B, 10% glycerol. C, 0.06% vitamin C + 0.05% niacinamide. D, 10% glycerol + 0.06% vitamin C + 0.05% niacinamide.

Figure 6 showed that at phylum level, the dominant bacteria in liver of four treatment groups were Firmicutes, Proteobacteria, Bacteroidota and Actinobacteriota. The relative abundances of these dominant bacteria in livers of pigs from groups A, B, C and D were 38.30, 54.47, 19.80 and 35.69%, respectively, for Firmicutes, were 36.21, 26.83, 49.08 and 27.52%, respectively, for Proteobacteria, were 14.31, 9.51, 25.03 and 19.73%, respectively, for Bacteroidota, were 7.23, 6.30, 3.20 and 5.50%, respectively, for Actinobacteriota. The first dominant phylum bacteria in livers were Firmicutes for pigs from groups A, B and D, but were Proteobacteria for pigs from group C. At genus level, the dominant bacteria in livers were *Stenotrophomonas* (20.60%), *Chryseobacterium* (7.31%), *Staphylococcus* (6.37%), *Clostridium_sensu_stricto_1* (5.93%), *Terrisporobacter* (4.68%) and *Delftia* (3.04%) for pigs from group A, were *Streptococcus* (6.81%), *Salinivibrio* (6.06%), *Clostridium_sensu_stricto_1* (4.83%), *Terrisporobacter* (4.61%), *Escherichia-Shigella* (4.54%) and *Stenotrophomonas* (4.35%) for pigs from group B, were *Stenotrophomonas* (36.26%), *Chryseobacterium* (11.19%), *Delftia* (5.43%), *Salinivibrio* (2.34%), *Blautia* (1.67%) and *Streptococcus* (1.37%) for pigs from group C, were *Stenotrophomonas* (14.41%), *Chryseobacterium* (5.99%), *Delftia* (3.54%), *Streptococcus* (3.21%), *Clostridium_sensu_stricto_1* (3.18%) and *Salinivibrio* (2.37%) for pigs from group D. The first dominant genus bacteria in livers were *Stenotrophomonas* for pigs from group A, C and D, but were *Streptococcus* for pigs from group B.

### 3.4 Differential bacterial composition in the liver of pigs

**Figure 7:**
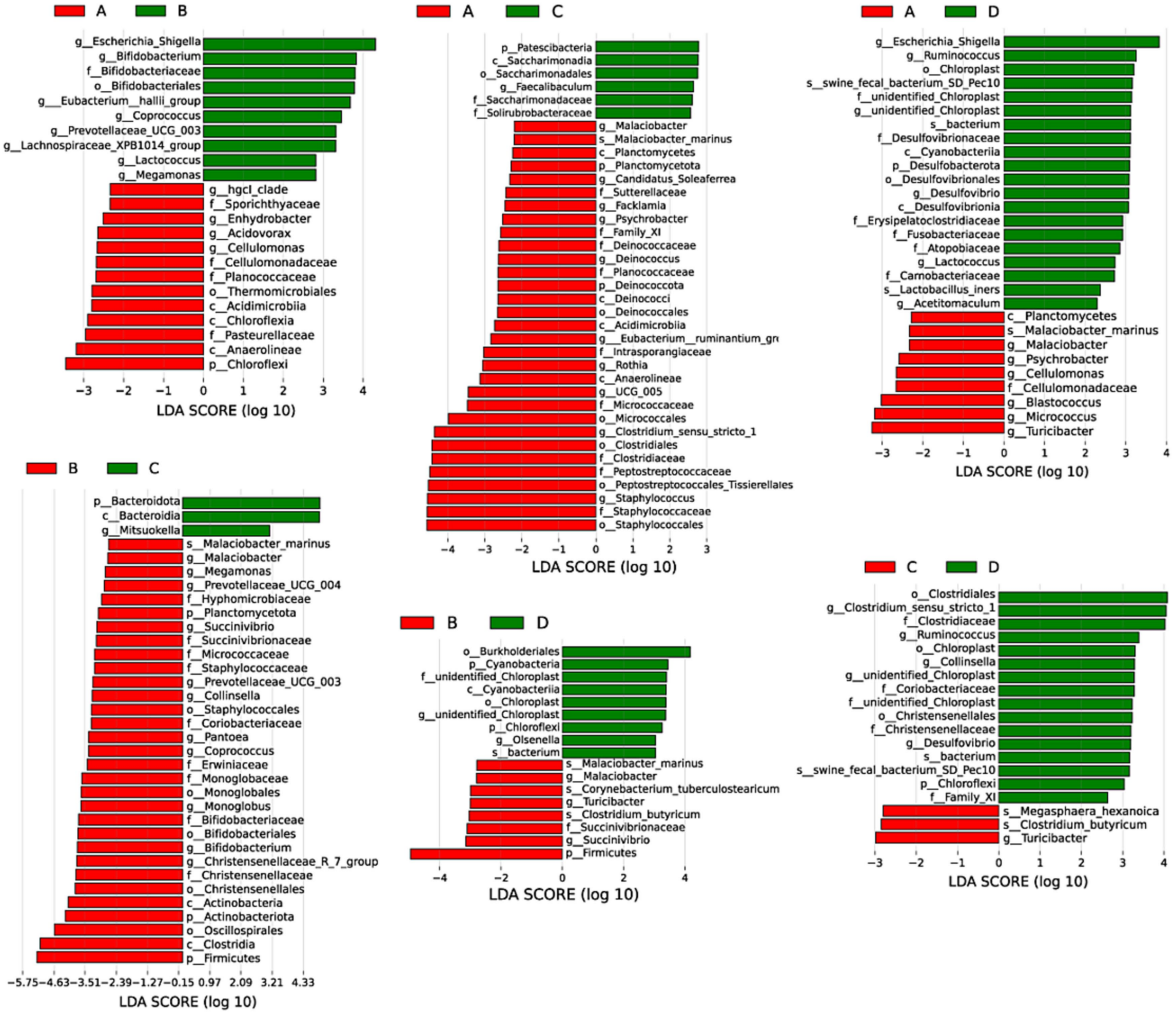
Differential bacterial composition of livers between groups. A, 0% glycerol + 0% vitamin C + 0% niacinamide. B, 10% glycerol. C, 0.06% vitamin C + 0.05% niacinamide. D, 10% glycerol + 0.06% vitamin C + 0.05% niacinamide.

LEfSe analysis was performed to discriminate the differential bacteria with significant difference between groups with the standards of LDA score > 2.0 and *p* < 0.05. Results in Figure 7 presented that compared with pigs from group A, pigs from group B had higher (*p* < 0.05) relative abundances of genus *Escherichia_shigella*, *Bifidobacterium*, *Eubacterium_hallil_group*, *Coprococcus*, *Prevotellaceae_UCG_003*, *Lachnospiraceae_XPB1014_group*, *Lactococcus* and *Megamonas*, lower (*p* < 0.05) relative abundances of genus *hgcl_clade*, *Enhydrobacter*, *Acidovorax*, *Cellulomonas*, *Planococcaceae* and phylum Chloroflexi in liver. Pigs from group C had higher (*p* < 0.05) relative abundances of genus *Faecalibaculum*, *Saccharimonadaceae* and phylum Patescibacteria, lower (*p* < 0.05) relative abundances of genus *Malaciobacter*, *Candidatus_Soleaferrea*, *Facklamia*, *Psychrobacter*, *Deinococcus*, *Eubacterium_ruminantium_gn*, *Rothia*, *UCG_005*, *Clostridium_sensu_stricto_1*, *Staphylococcus*, and phylum Planctomycetes, Deinococcota in livers than pigs from group A. The relative abundances of genus *Escherichia_Shigella*, *Ruminococcus*, *Unidentified Chloroplast*, *Desulfovibrio*, *Lactococcus*, *Acetitomaculum* and phylum Desulfobacterota were higher (*p* < 0.05) but the relative abundances of genus *Malaciobacter*, *Psychrobacter*, *Cellulomonas*, *Blastococcus*, *Micrococcus*, *Turicibacter* were lower (*p* < 0.05) when compared pigs from group D with pigs from group A. Compared with pigs from group B, pigs from group C had higher (*p* < 0.05) relative abundances of genus *Mitsuokella* and phylum Bacteroidota, but lower (*p* < 0.05) relative abundances of genus *Malaciobacter*, *Megamonas*, *Prevotellaceae_UCG_004*, *Succinivitrio*, *Prevotellaceae_UCG_003*, *Collinsella*, *Pantoea*, *Coprococcus*, *Monoglobus*, *Bifidobacterium*, *Christensenellaceae_R_7_group*, and phylum Planctomycetota, Actinobacteriota and Firmicutes in liver. Pigs from group D had higher (*p* < 0.05) relative abundances of genus *Unidentified_Chlorofleil*, *Olsenella* and phylum Cyanobacteria and Chloroflexl, but lower (*p* < 0.05) relative abundances of genus *Malaclobacter*, *Turicibacter*, *Succinivibrio*, and phylum Firmicutes in livers than pigs from group B. Pigs from group D had higher (*p* < 0.05) relative abundances of genus *Clostridium_sensu_stricto_1*, *Ruminococcus*, *Collinsella*, *unidentified_Chloroplast*, *Desulfovibrio*, phylum Chloroflexi, lower (*p* < 0.05) relative abundances of genus *Turicibacter* in liver than pigs from group C.

### 3.5 Differentially expressed genes, Kyoto encyclopedia of genes and genomes pathway enrichment and qPCR validation

**Figure 8:**
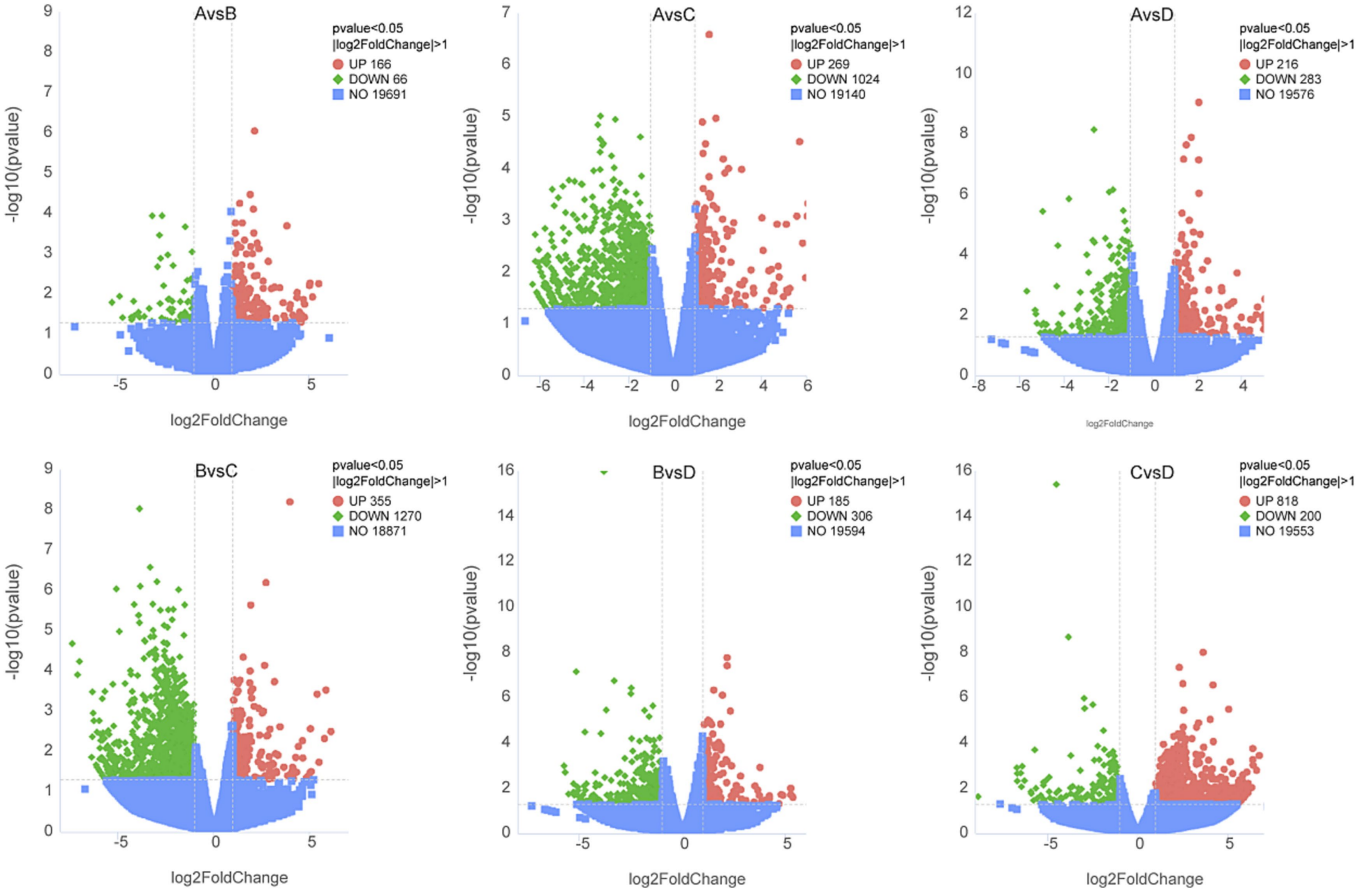
Volcano plot of differentially expressed genes in livers between groups. A, 0% glycerol + 0% vitamin C + 0% niacinamide. B, 10% glycerol. C, 0.06% vitamin C + 0.05% niacinamide. D, 10% glycerol + 0.06% vitamin C + 0.05% niacinamide. The abscissa represents the fold changes in gene expression. The ordinate represents the statistical significance of the variations in gene expression. The red dots represent significantly differentially expressed genes.

**Figure 9:**
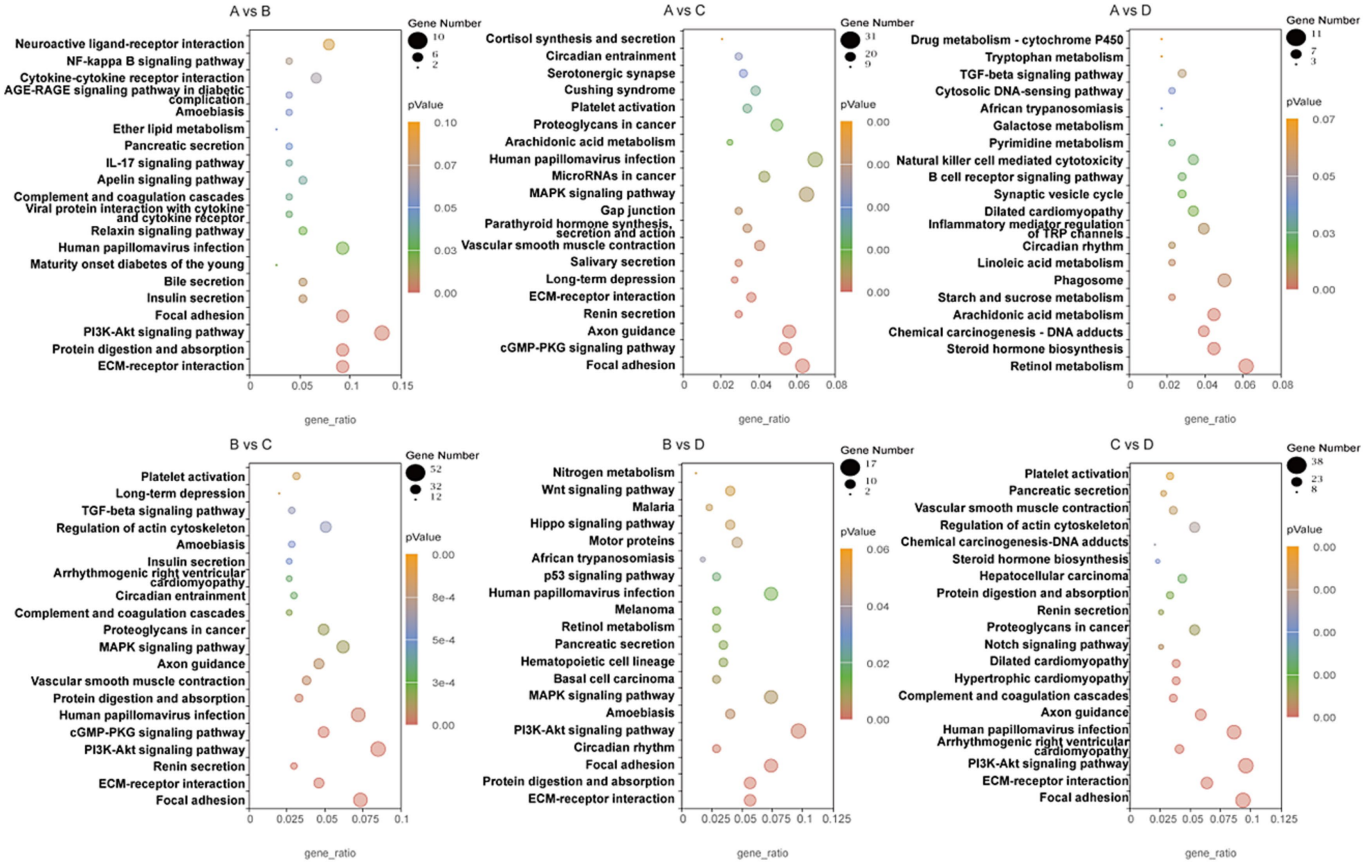
KEGG pathways of differentially expressed genes in livers between groups. A, 0% glycerol + 0% vitamin C + 0% niacinamide. B, 10% glycerol. C, 0.06% vitamin C + 0.05% niacinamide. D, 10% glycerol + 0.06% vitamin C + 0.05% niacinamide.

RNA sequencing was conducted to trace transcriptome changes, |log2(FoldChange)| ≥ 1 and *p <* 0.05 were used to screen the differentially expressed genes of livers between groups. Figure 8 showed that the numbers of differential genes with significant difference were 232 (166 up, 66 down) between groups A and B, 1293 (269 up, 1,024 down) between groups A and C, 499 (216 up, 283 down) between groups A and D, 1625 (355 up, 1,270 down) between groups B and C, 491 (185 up, 306 down) between groups B and D, 1018 (818 up, 200 down) between groups C and D, respectively. The differentially expressed genes between groups were mapped into KEGG biochemical pathways, and the 20 most abundant KEGG pathways between groups are presented in Figure 9.

Thirteen KEGG pathways were related to the nutrition metabolism and disease occurrence in liver, including protein digestion and absorption, bile secretion, viral protein interaction with cytokine and cytokine receptor, PI3K-Akt signaling pathway, steroid hormone biosynthesis, arachidonic acid metabolism, starch and sucrose metabolism, linoleic acid metabolism, focal adhesion, phagosome, inflammatory mediator regulation of TRP channels, hematopoietic cell lineage and circadian rhythm. Differentially expressed genes involved in inflammation and lipid metabolism in these KEGG pathways are listed in Table 4.

**Table 4:** Genes regulating inflammation and lipid metabolism.

Items	Gene_name	Gene expression
Group A-Group B	*DEGS2*(delta 4-desaturase, sphingolipid 2)	down
	*EDAR*(ectodysplasin A receptor)	down
	*CXCL2*(C-X-C Motif Ligand 2)	up
	*TNFSF14*(Tumor Necrosis Factor Ligand Superfamily, Member 14)	up
	*SLC7A11*(Solute Carrier Family 7, Member 11)	down
Group A-Group C	*IL18*(interleukin 18)	down
	*DGKH*(Diacylglycerol Kinase Eta)	up
	*DGKE*(diacylglycerol kinase epsilon)	up
	*LIF*(Leukemia Inhibitory Factor)	up
	*GPM6A*(glycoprotein M6A)	up
	*PAG1*(phosphoprotein membrane anchor with glycosphingolipid microdomains 1)	up
	*MBOAT2*(membrane bound O-acyltransferase domain containing 2)	up
	*GLTPD2*(glycolipid transfer protein domain containing2)	down
	*SLC7A11*(Solute Carrier Family 7, Member 11)	up
Group A-Group D	*CRP*(C-reactive protein)	down
	*TNFSF10*(Tumor Necrosis Factor Ligand Superfamily, Member 10)	up
	*GALK1*(Galactokinase 1)	down
	*CRY1*(cryptochrome circadian clock 1)	up
	*SLC7A11*(Solute Carrier Family 7, Member 11)	up
Group B-Group C	*GLTPD2*(glycolipid transfer protein domain containing2)	down
	*PLPP6*(phospholipid phosphatase 6)	down
	*GALK1*(Galactokinase 1)	down
	*DGKH*(Diacylglycerol Kinase Eta)	up
	*MBOAT2*(membrane bound O-acyltransferase domain containing 2)	up
	*GRAMD1B*(GRAM domain containing 1B)	up
	*PAG1*(Phosphoprotein associated with glycosphingolipid-enriched microdomains 1)	up
	*LDAH*(Lipid droplet-associated hydrolase)	up
	*LIF*(leukemia inhibitory factor)	up
	*TNFRSF11B*(TNF receptor superfamily member 11b)	up
	*SLC7A11*(Solute Carrier Family 7, Member 11)	up
Group B-Group D	*CRP*(C-reactive protein)	down
	*GALK1*(Galactokinase 1)	down
	*CCL2*(chemokine (C-C motif) ligand 2)	up
	*LIF*(leukemia inhibitory factor)	up
	*CCL21*(chemokine (C-C motif) ligand 2)	up
	*TPD52L1*(tumor protein D52-like 1)	up
	*TNFAIP8*(Tumor Necrosis Factor Alpha-induced Protein 8)	up
	*CRY1*(cryptochrome circadian clock 1)	up
	*SLC7A11*(Solute Carrier Family 7, Member 11)	up
Group C-Group D	*RASGRP3*(Ras guanyl nucleotide releasing protein 3)	down
	*DGKH*(Diacylglycerol Kinase Eta)	down
	*ABHD2*(abhydrolase domain containing 2)	down
	*DGKI*(diacylglycerol kinase iota)	down
	*MBOAT2*(membrane bound O-acyltransferase domain containing 2)	down
	*SLC7A11*(Solute Carrier Family 7, Member 11)	down
	*CXCL9*(C-X-C motif ligand 9)	up
	*GLTPD2*(glycolipid transfer protein domain containing2)	up
	*GALK1*(Galactokinase 1)	down

**Figure 10:**
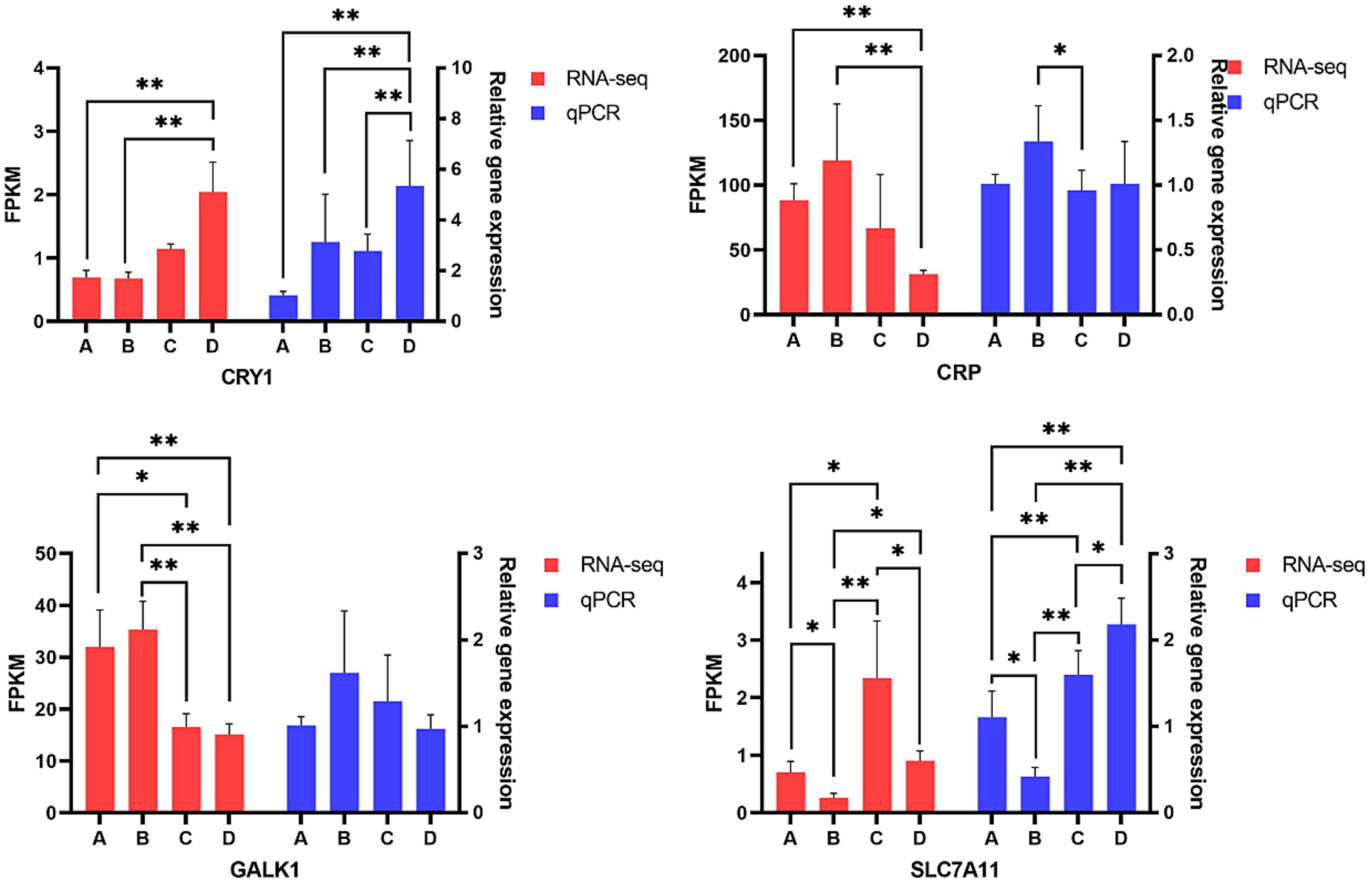
Comparison of genes expression level in liver. A, 0% glycerol + 0% vitamin C + 0% niacinamide. B, 10% glycerol. C, 0.06% vitamin C + 0.05% niacinamide. D, 10% glycerol + 0.06% vitamin C + 0.05% niacinamide. *p < 0.05, **p < 0.01.

In order to validate the accuracy of gene expression level generated by RNA-Sequencing, four differentially expressed genes including *CRP*, *CRY1*, *GALK1* and *SLC7A11*, which were transcription factors related to inflammation and lipid metabolism, were selected to perform qPCR assays. The results of Figure 10 indicated the trends of relative expression levels of *CRP*, *CRY1*, *GALK1* and *SLC7A11* of qPCR were similar to that calculated by transcriptome sequencing, confirming the reliability of transcriptome data. qPCR data showed that pigs from group D had higher *CRY1* expression in liver than pigs from groups A, B and C (*p* < 0.01), respectively. Pigs from group B had higher *CRP* expression in liver than pigs from group C (*p* < 0.05). There was no significant difference in the relative abundance of *GALK1* of livers among four groups but pigs from group B had the numerically highest *GALK1* expression when compared to pigs from the other three groups. Pigs from group B had the lowest *SLC7A11* expression but pigs from group D had the highest *SLC7A11* expression in liver, pigs from group B had lower *SLC7A11* expression than pigs from group A (*p* < 0.05), pigs from groups C and D had higher *SLC7A11* expression in liver than pigs from group B (*p* < 0.01), respectively.

## 4 Discussion

The liver plays important roles in keeping health and high production of animals, diet ingredients can influence the health and functions of liver via regulating energy metabolism, fat deposition, inflammation and microbiota composition (35, 36). Glycerol is often used in the diet, it can yield energy through the glycolytic and tricarboxylic acid pathways because it is an intermediate in the lipogenesis and gluconeogenesis pathways (37). Long-term inappropriate consumption of food containing glycerol may lead to liver diseases by changing the growth and histomorphology of liver with the accumulation of fat and trace elements. Studies indicated that the index or weight of liver can be altered by feeding glycerol to animals. It is reported that liver index significantly increased when increasing glycerol level (2.5, 5.0, 7.5, 10%) in the diet of broilers from day 1 to day 21 (38), and 3% glycerol addition reduced the liver weight of 40-d-old broilers (39), however, Topal and Ozdogan (40) reported dietary addition of glycerol at 5 or 8% had no effects on liver size of broilers. In addition, dietary glycerol supplementation also had influences on liver histomorphology, supplementing glycerol to postpartum goats improved liver morphology (41), but the liver histomorphology of growing-finishing pigs offered a corn-soybean meal-based diets was not influenced by glycerol addition whenever at levels of 5% or 10% (42). Results of this study showed that dietary glycerol supplementation at level of 10% to the diet of growing-finishing pigs numerically reduced liver index, increased liver fat content and developed partially hepatic steatosis along with small vacuoles in the cytoplasm. Hepatic steatosis is accompanied by the increase of fat content and the formation of vacuoles of different sizes in liver cells, which can lead to dysfunction, necrosis, fibrosis, or cirrhosis of liver (43). Previous studies showed that increasing niacinamide supplementation (10, 30, 1,000 mg/kg diet) recovered liver morphology to normal structure by alleviating hepatocyte steatosis (44). Niacinamide addition at high levels has potential risk to liver, because water drinking with niacinamide at 1% or 0.25% had a trend to reduce the liver size of mice (45) and supplementation of niacinamide with 500 mg/kg BW to healthy animals induced hepatotoxicity (22). Hepatotoxicity of niacinamide addition in this study did not occur, it might be the hepatoprotective effect of vitamin C, Abd-Allah et al. reported that administrating niacinamide together with vitamin C can reverse hepatotoxicity (30). Skat-Rørdam et al. also demonstrated that supplementing vitamin C at a level of 2000 mg/kg to high fat diet decreased liver cell ballooning of rats (46).

Hepatic steatosis, iron overload and pathogen infection can cause various liver diseases, including metabolic-associated fatty liver conditions such as steatohepatitis and fatty liver. The former is defined as the presence of macrovesicular steatosis in addition to hepatocyte ballooning degeneration, lobular inflammation, and/or fibrosis, the latter is characterized as macrovesicular steatosis without ballooned hepatocytes (47–49). Steatohepatitis has been shown to be associated with inflammatory factors, especially pro-inflammatory cytokines such as TNF-*α*. Eltahir et al. reported that glycerol injection increased serum TNF- *α* level of rats (50). Dietary vitamin C addition can promote iron absorption from gut content (51), and iron overload in liver can cause inflammation, promote hepatic steatosis and fibrosis, or trigger ferroptosis of hepatocyte through Fenton reaction and reactive oxygen species (ROS) accumulation (52). Vitamin C addition at the level of 2000 mg/kg diet significantly decreased inflammation in hepatic tissue of rats subjected to high fat diet (46). Bae et al. also found that vitamin C addition decreased the TNF-*α* level of liver tissue in Gulo(−/−) mice maintained on the vitamin C supplemented water (3.3 g ascorbate/L) (53). Lots of studies demonstrated that niacinamide has anti-inflammatory property (54, 55), and niacinamide addition to the diet can increase IL-10 level but decrease TNF-α level of ApoE-deficient mice (45). TNF-α is the cytokine responsible for steatohepatitis progression, patients with steatohepatitis have higher TNF-α levels, which play an important role in hepatic fibrosis (56), insulin resistance and type 2 diabetes (57, 58). Results of this study indicated that pigs only supplemented with 0.06% vitamin C and 0.05% niacinamide mixture had higher level of ferrous ion in liver, but had less steatosis and lower concentration of TNF-α in liver compared with pigs offered with 10% glycerol alone. This may be due to the antioxidant function of vitamin C, as it can reduce the production of ROS by alleviating ferroptosis, thereby reducing the damage of iron ion accumulation to the liver, because high dietary vitamin C intake can improve glucose metabolism and liver function by reducing iron accumulation (59).

Liver also harbors microbiota and the microbial composition has impacts on the health and functions of liver. It is reported that cattle with liver abscess had the most abundant Fusobacteriota and Bacteroidota, *Fusobacterium* and *Bacteroides* in liver tissue (60–63). Bacteroidota and *Faecalibacterium* were significantly expressed in both fatty liver and steatohepatitis patients, but *Megamonas* and *Fusobacterium* were also found in the steatohepatitis patients (64). *Escherichia_Shigella* can induce hepatic steatosis, ballooning, hepatic inflammation and fibrosis by inhibiting the expression of hepatic peroxisome proliferator activated receptor PPAR*α* (65). *Lachnospiraceae_XPB1014_group* is positively correlated with TNF-α, liver index and liver weight, respectively (66). *Desulfobacterotam*, *Lactococcus* and *Faecalibaculum* can induce inflammation (66–68). *Megamonas* is also known to enrich in inflammatory diseases such as non-alcoholic fatty liver disease (69–71). It is reported vitamins can affect the community structure, richness and diversity as well as the relative abundance of microbiota in hepatic tissue of animals (72), but prenatal supplementation with vitamin A, D and E had no significant effects on the composition and relative abundance of microbiota in hepatic tissue of calves (72). Results of this study demonstrated that single addition of 10% glycerol significantly increased the relative abundances of *Escherichia_shigella*, *Lachnospiraceae_XPB1014_group*, *Lactococcus* and *Megamonas* in liver tissue, which were the contributors for the increased TNF-*α* level. The addition of 0.06% vitamin C and 0.05% niacinamide mixture significantly increased the relative abundance of *Faecalibaculum* in liver, but there was no strong positive correlation between *Faecalibaculum* and TNF-α (66), this might be the reason why the TNF-α level was not significantly increased in liver tissue of pigs supplemented with mixture of 0.06% vitamin C and 0.05% niacinamide.

Gene expression in liver tissue also has influences on liver health and functions. The proinflammatory cytokines can be inhibited by *CRY1* gene expression (73) or enhanced by *CRP* gene expression (74). It is reported that CRP plays important roles in the production of cytokines, particularly interleukin-6 and tumor necrosis factor-*α*, the elevated CRP gene expression can increase the concentration of TNF-α (75). In this study, the highest TNF-α concentration in the liver of pigs supplemented with only 10% glycerol should be contributed to the highest CRP gene expression. Pigs supplemented with 10% glycerol together with 0.06% vitamin C and 0.05% niacinamide had the similar CRP gene expression but had the lowest CRP concentration in liver, this could be explained by the highest CRY1 expression in the liver of pigs. *GALK1* gene is involved in fat deposition (76), and *SLC7A11* gene has the function of inhibiting ferroptosis in liver cells (77). Data of this study indicated that addition of 10% glycerol increased the expression levels of gene *CRY1*, *CRP* and *GALK1* but decreased the expression level of gene *SLC7A11*, the decreased *SLC7A11* expression should be one of the contributors to increase TNF-α level of liver by increasing ferroptosis. Supplementation of 0.06% vitamin C and 0.05% niacinamide mixture enhanced *SLC7A11* gene expression, the elevated *SLC7A11* gene expression might be the important factor why the mixture of 0.06% vitamin C and 0.05% niacinamide increased liver iron content without causing liver organ damage.

## 5 Study limitations and future directions

An outbreak and rapid spread of infectious disease occurred in some experimental pigs approaching the end of experiment. In order to prevent the spread and transmission of this infectious disease, experimental pigs had to be transported out for harmless treatment. Prior to being transported away of pigs, we did not have time to collect sufficient samples, only three pigs without disease symptoms were selected from each group for slaughter to collect liver and pancrea samples, resulting in an insufficient sample size of liver and pancrea. Data of this preliminary experiment indicated that dietary addition of glycerol together with or without mixture of vitamin C and niacinamide had different effects on the health and function of the liver and pancreas of growing-finishing pigs. In the near future, larger scale experiment will be conducted together with pig farms to harvest sufficient samples of liver, pancrea and gut digesta to verify the current data, assay the long-term toxicity and disclose the causality between microbiota shifts and hepatic inflammation.

## 6 Conclusion

Dietary addition with 10% glycerol only increased TNF-*α* concentration, steatosis, relative abundances of *Escherichia_shigella*, *Lachnospiraceae_XPB1014_group*, *Coprococcus*, *Lactococcus* and *Megamonas*, but decreased SLC7A11 expression in liver of growing-finishing pigs. However, supplementation of the mixture with 0.06% Vitamin C and 0.05% niacinamide alleviated steatosis and inflammation in liver of growing-finishing pigs fed diets containing 10% glycerol by reducing the numbers of harmful microbiota and increasing the expressions of CRY1 and SLC7A11.

## Data Availability

The datasets presented in this study can be found in online repositories. The names of the repository/repositories and accession number(s) can be found in the article/supplementary material.
